# Notes and News

**Published:** 1894-11-17

**Authors:** 


### Notes and News.

Collected and Communicated.

It is proposed to make an addition to the Darlington Hospital. This will specially consist of a children's wing, a feature which will be very necessary if the arrangement to close the Children's Hospital in Victoria Road is carried out. Appeals to further the project are to be made in the town and district.

We congratulate Mr. Howgrave Graham on the success of his efforts in behalf of his hospital, the Hospital for Paralysis and Epilepsy, Regent's Park. We mentioned some time ago that Mr. Howgrave Graham was presenting the proceeds from the sale of his attractive setting of "Break, Break, Break," to the hospital, and are now glad to state that the amount thus received has already exceeded £30. We feel sure that as the song is more widely known so will its popularity increase, to the benefit of the hospital of which Mr. Graham is the able secretary.

The directors of the Dundee Infirmary have made the inquiries they instituted at the various centres of medical education as to the custom of holding classes of male and female students together. They find that at Belfast only is mixed instruction given. At the meeting held to decide the question, the directors resolved that mixed classes should not be sanctioned, but they have deputed the medical superintendent and teaching staff to prepare a scheme whereby lady students can receive separate instruction. This being done, they will consider the question again.

The abuse of the out-patients' department is a blot on our hospital system, and one that it is difficult to remove. It is a sore point with all conscientious secretaries. Quite recently we heard of a secretary who, receiving no support from his committee in checking the evil, determined to see what could be done by appealing to the patients themselves. Accordingly he appeared frequently in the out-patients' department, and put the matter of contributing, each according to his means, before them, with the result that the voluntary contributions in the collecting box were at once quadrupled. Something evidently may be done by educating the out-patient.

The Hospital Sunday collection Fund in Birmingham has reached over £4,000. The day was inclement, and the attendance at the churches was not numerous. Last year the 67 contributing congregations produced £2,725, and the result this year is therefore most encouraging.

Dr. C. Albert Hingston, a most generous philanthropist has just presented a new convalescent home to Plymouth and the neighbourhood, situated at Crown Hill. The institution, which will contain twenty-two patients, eleven of each sex, has cost £4,000. At the opening ceremony the Misses Prideaux made a donation of £100, while another friend presented a piano.

The new buildings at the Medical School, Owens College, Manchester, are completed, and have been opened by the Duke of Devonshire. They comprise lecture theatres, class-rooms, laboratories, and administrative-rooms, the whole addition being larger than the existing Medical College, which was already a very excellently equipped building. At the time when the Medical College was incorporated with the Owens College 99 students were attending. This was in 1871. In 1891 the students numbered 423. In providing the new buildings the council have taken into consideration the increased requirements of medical education, and are confident that as it is now constituted the Manchester school affords all needed opportunities for education and investigation to practitioners, who will receive every assistance in their work.

The drawbacks to the barrack-school system have been widely ventilated, and it will be interesting to note how far the objections raised to it will be allowed to influence charitable institutions not under State control. For instance, everybody has heard of the Kilburn Orphanage, which is such an important feature of the Church Extension Association; but does anyone reflect on the future of these massed-together children? Fifty little ones sleep in one great dormitory, each being enclosed in a cell of open iron-work, the door secured on the outside. If the moral influence of the home is only maintained by iron bars, what protection will it secure to the orphan girls when they try their wings in the great world which lies beyond the restraining cage?

Dr. Edward Magennis delivered an excellent address before the Congress of the British Institute of Public Health last July on hygiene in the schoolroom, in which was discussed the important questions of the construction, ventilation, lighting, and heating of the schoolroom. Although schoolrooms have improved during the last few years, still much is required before we fulfil our duties to the young during their growing years. For instance, in most of our public elementary schools somewhere about 120 cubic feet of air space is provided, whereas each child ought to be supplied with 400 cubic feet, and until this is carried out school mortality must continue unnecessarily and unwarrantably high. Further, we shall never rest content until there is a Minister of Public Health, who will organise an inspection of all schools throughout the country, for some of the worst offenders in school hygiene are schools of the highest grade.

